# Higher Acceptance of Videotelephonic Counseling Formats in Psychosomatic Medicine in Times of the COVID-19 Pandemic

**DOI:** 10.3389/fpsyt.2021.747648

**Published:** 2021-10-27

**Authors:** Jacqueline Lohmiller, Norbert Schäffeler, Stephan Zipfel, Andreas Stengel

**Affiliations:** ^1^Department of Psychosomatic Medicine and Psychotherapy, University Hospital Tübingen, Tübingen, Germany; ^2^Department for Psychosomatic Medicine, Charite-Universitätsmedizin Berlin, Charité Center for Internal Medicine and Dermatology, Corporate Member of Freie Universität Berlin, Humboldt-Universität zu Berlin and Berlin Institute of Health, Berlin, Germany

**Keywords:** acceptance, assessment, office counseling, telephone counseling, video counseling

## Abstract

**Background:** Due to the COVID-19 pandemic, the healthcare system in general and psychosomatics in particular were forced to change counseling-specific services and break up established structures. At the beginning of 2020, phone as well as videotelephonic counseling options had to be quickly established.

**Methods:** Patients (*n* = 278) of the department of psychosomatic medicine and psychotherapy at the University Hospital Tübingen were asked to complete an *ad hoc* questionnaire to assess the acceptance of the counseling format following each counseling session (office, phone, video) in the period between July 2020 and February 2021.

**Results:** Satisfaction and acceptance of the three counseling formats (office, phone, video) were rated (1–6) on average as “good” to “very good” in the three subgroups (1.5 ± 0.9). Likewise, the “therapeutic relationship” scored high in all three subgroups in terms of establishing a strong therapeutic relationship (4.4 ± 1.5). “Hurdles” were rated as low and tolerable (1.8 ± 1.3). The global assessment of therapeutic contact was significantly better in the video group compared to phone and office consultation (*p* < 0.05). Predictor analyses showed that there was an influence of age, but not gender, on the acceptance of digital counseling formats in the present sample [*F*_(1, 277)_ = 4.50, *p* = 0.04].

**Discussion & Conclusion:** Digital consultation formats were perceived by patients as promising addition to the classic face-to-face setting. Digital formats (phone, video) were not generally preferred to face-to-face counseling, but especially video counseling was accepted and perceived with great satisfaction and acceptance. Accordingly, the additional use of digital counseling formats, especially video-telephony, could be an opportunity to enrich the existing structures also after the pandemic.

## Introduction

The COVID-19 pandemic posed new challenges for the healthcare system in general and psychosomatics in particular (7). The ubiquitous “subject” of digitization came into focus and opened up alternative care pathways (8). The pandemic imposed the compelling need to establish phone as well as video counseling systems to ensure consultation as well as continuity of care (9, 10). Our psychosomatic outpatient clinic acted accordingly to the pandemic circumstances in spring 2020 and introduced phone as well as video counseling in addition to the well-known office counseling (11). Because of the lockdown and all the associated restrictions, the online-based counseling setting may provide an alternative to the traditional office visit. The discussion regarding the advantages and disadvantages of an online-based therapeutic care structure is controversial (5, 12, 13). The main point of contention manifests with regard to the feasibility of psychotherapeutic intervention measures (14, 15). The question arises whether and to what extent a therapeutic relationship can be established in the context of phone or video counseling. The impact of the therapeutic relationship on therapy outcomes is widely recognized and directs focus to the communication channel through which relationship building occurs. Numerous studies demonstrate effective therapeutic relationship building in the online setting (2, 15–17), but the question arises as to whether the hurdles of online-based procedures counteract the benefits of phone/video-based outpatient psychosomatic consultation.

Given the need to maintain therapeutic counseling services in the context of the COVID-19 pandemic, the National Association of Statutory Health Insurance Physicians facilitated the restructuring of the traditional on-site counseling setting. The use of tele-medicine was on the rise and scored high in terms of flexibility, time efficiency, mobility-related hurdle reduction (as the calls can take place at home without the need to take e.g., public transportation and to meet other people), and cost-effectiveness.

To record the acceptance of the offered additional phone and videotelephonic counseling media the subject areas “patient characterization” (descriptive data: gender, age, type of contact, frequency of contact, occasion of contact, treating therapist, etc.), “assessment of therapeutic contact,” “therapeutic relationship,” and “hurdles” were recorded by means of an *ad hoc* questionnaire. Using these data the following questions were investigated after individual as well as group sessions with patients in psychosomatic medicine: How do patients evaluate tele-based counseling compared to counseling in the office? Does satisfaction differ depending on the counseling setting? Do age and gender have an influence on the acceptance of the different counseling formats?

## Methods

### Procedure

The survey of the acceptance of the offered counseling media in the psychosomatic outpatient clinic at the University Hospital in Tübingen was conducted by means of an explorative online survey and took place in the period from July 2020 to February 2021. All therapists were employees of the psychosomatic outpatient clinic, University Hospital Tübingen. All patients who attended an individual or group consultation at the psychosomatic outpatient clinic at the University Hospital in Tübingen were offered the opportunity to participate in the survey. The patients, who had given their written consent after detailed information and explanation, participated in the anonymous data collection. The questionnaire was handed out in digital form by e-mail after each consultation by one of the three counseling formats (office, phone, video). All patients who were offered an appointment were also sent the questionnaire. Accordingly, all patients were given the opportunity to participate in the survey. The consultation could be an initial interview contact at the psychosomatic outpatient clinic (counseling interview, 35.3%; anamnesis interview, 29.5%; planning interview for admission in day clinic/ward, 30.6%), or could occur as part of an ongoing consultation at the psychosomatic outpatient clinic (individual therapy interview, 1.8%; group therapy interview, 2.9%). All patients were offered the option of telephone or video consultation. Depending on pandemic restrictions, office consultation could sometimes not be offered. The study was approved by the local ethics committee (458/2020BO).

### Sample

A total of 278 patients (182 female, 95 male, 1 diverse = non-binary gender) participated in the survey to record their assessment of and satisfaction with the three counseling formats offered. The following analyses are based on the descriptive and psychometric data collected using a specially designed questionnaire.

### Survey

In total, the questionnaire battery used consisted of 4 subject areas “patient characterization,” “assessment of therapeutic contact,” “therapeutic relationship,” and “hurdles.” The “patient characterization” had 7 nominally (gender, age, type of contact, frequency of contact, reason for contact, treating therapist, contact options offered) and 3 metrically (global judgment conversational contact, overall impression, reasons for choice of contact type) scaled items. The “assessment of therapeutic contact” consisted of 12 items recorded on 6-point Likert scales (with items such as personal to impersonal, friendly to unfriendly, appreciative to not appreciative, etc.). The “therapeutic relationship” subject area encompassed 11 items recorded on a 6-point Likert scale with the categories 1 = very inapplicable to 6 = very applicable derived from the German version of the helping alliance questionnaire (6). Lastly, the “hurdles” comprised 5 items to be answered on a 6-point Likert scale (with categories 1 = strongly disagree to 6 = strongly agree). In addition, 5 free-text items were used to collect open-ended responses to questions regarding reasons for choosing the type of contact and on feedback (“Is there anything you particularly liked or disliked about the counseling/counseling center?”).

The construction of the “assessment of therapeutic contact” subject area was based on the predictors “global judgment” and “experiencing the contact”. The “therapeutic relationship” subject area inquired about the assessment of the relationship with the therapist. Lastly, the “hurdles” recorded the subjective perception regarding the “technique” or the “framework conditions” and, if applicable, the safety instructions (e.g., wearing a mask, disinfection, distance, etc.).

### Statistical Analyses

The analyses were performed using the statistical software IBM SPSS Statistics (version 27, IBM Corp, 2017). Sociodemographic characteristics were analyzed descriptively. No outliers were found in the analysis of the standardized residuals. In addition, we checked for violations of the assumptions of collinearity, independent error, normal distribution, homoscedasticity, and linearity. In this context, all assumptions were met. A χ^2^-test for association was conducted between gender, respectively, age and assessment. In addition, ANOVAs were used to test whether age and gender had an impact on the assessment. Hierarchical regression analyses were performed to examine the associations between age or gender and three dependent variables “assessment of therapeutic contact,” “therapeutic relationship,” and “hurdles.”

## Results

The study population consisted of 278 patients (182 female, 95 male, 1 diverse). The mean age was 31.5 years (range = 18–80 years). Of the 278 participants, 46.4% of counseling contacts occurred via the face-to-face office format, 9.7% via phone, and 43.9% via the video telephonic format (Table 1). Besides, 14% of the contacts were with a female physician and 16.9% with a male physician. 47.5% reported that the consultation took place with a female psychologist and 9.7% with a male psychologist. 7.6% conducted the consultation with a nurse and 4.3% did not recall the profession of the therapist (Table 1). Within the present sample, 35.3% indicated that the contact occurred as a counseling interview, and 29.5% answered that the contact occurred as a medical history interview. 1.8% categorized the contact occasion as an individual therapy session. In 2.9% the contact took place as a group therapy session and in 30.6% as a planning interview for admission (day clinic /ward, Table 1).

**Table 1:** Description of the study population (*n* = 278).

**Characteristic**		**Mean**	**%**
**Age**		31.5 (years)	
**Gender**			
	Male		17.0
	Female		83.0
**Contact type**			
	Office consultation		46.4
	Phone consultation		9.7
	Video consultation		43.9
**Treating therapist**			
	Doctor (f)		14.0
	Doctor (m)		16.9
	Psychologist (f)		47.5
	Psychologist (m)		9.7
	Nurse (f)		7.6
	Was not remembered (m)		4.3
**Contact occasion**			
	Counseling interview		35.3
	Anamnesis interview		29.5
	Individual therapy interview		1.8
	Group therapy interview		2.9
	Planning interview for admission (day clinic / ward)		30.6

**Figure 1:**
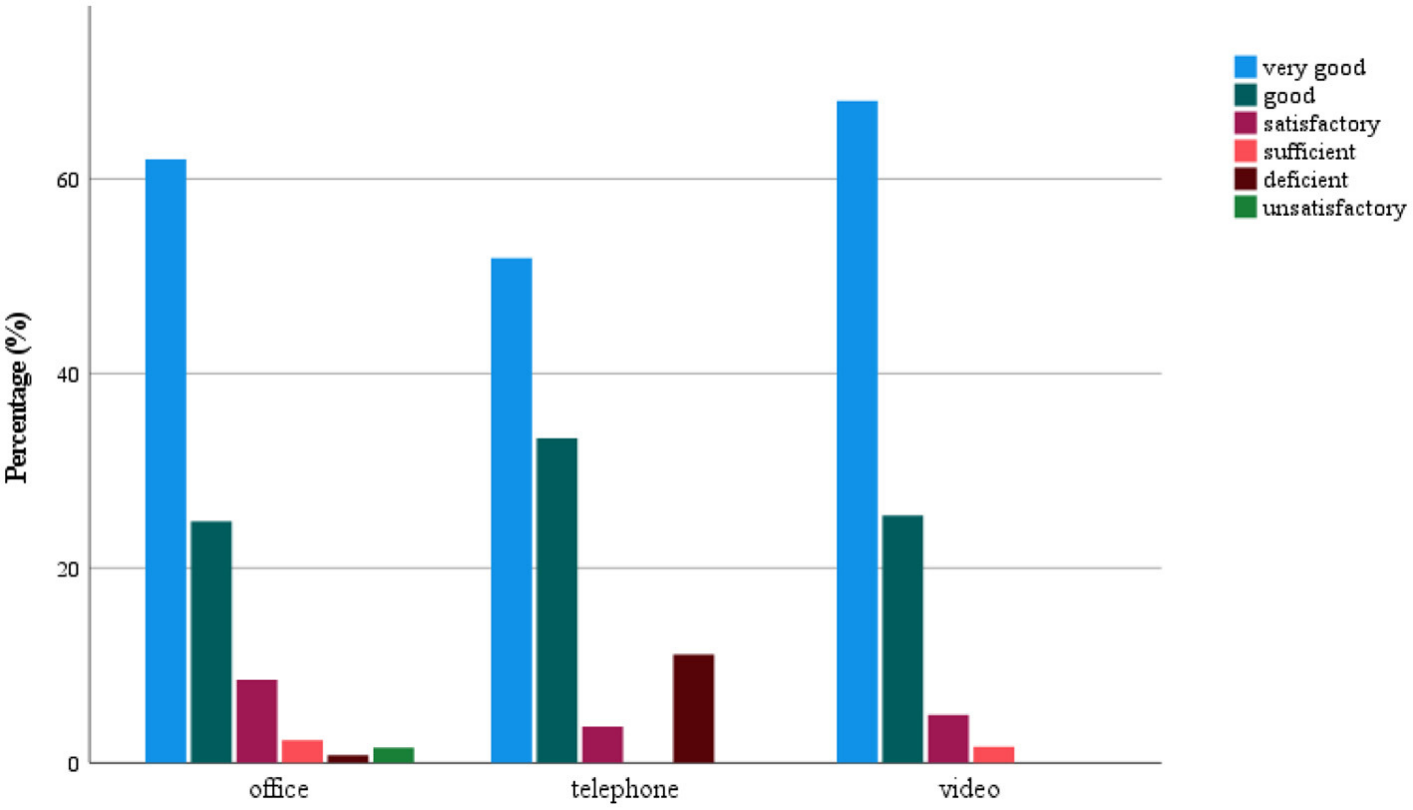
Satisfaction with the type of contact.

Satisfaction ratings comparing the three counseling formats showed on average a high to very high level of satisfaction (Figure 1). Contact during office counseling was rated as very good in 62%, good in 24.8%, satisfactory in 8.5%, sufficient in 2.3%, deficient in 0.8%, and unsatisfactory in 1.6% of cases. Phone contact was rated as very good in 51.9%, good in 33.3%, satisfactory in 3.7%, and deficient in 11.1% of cases. Lastly, video contact was rated very good in 68.0%, good in 25.4%, satisfactory in 4.9%, and sufficient in 1.6% of cases (Figure 1). Office as well as video counseling were rated very good to good on average and seemed to be preferred over phone counseling. Therefore, the next step was a detailed evaluation of the subject areas “assessment of therapeutic contact,” “therapeutic relationship,” and “hurdles.”

At this point, we have computed an analysis of variance. Within the “assessment of therapeutic contact” the item “global judgment” had a mean of 1.5 (*SD* = 0.9) in the total group. In the office subgroup, the *Mean* ± *SD* of this item was 1.6 ± 1.0, in the phone subgroup 1.9 ± 1.3, and in the video subgroup 1.4 ± 0.7 [*F*_(2, 275)_ = 3.39, *p* = 0.04; Table 2]. All other items rated ranged from *M* = 1.2–2.1, both for the overall group and within the three subgroups, i.e., all were rated between very good and good. Moreover, significant differences were found between the subgroups related to the items pleasantness [*F*_(2, 275)_ = 3.35, *p* = 0.04], friendliness [*F*_(2, 275)_ = 5.55, *p* = 0.004] and feeling comfortable [*F*_(2, 275)_ = 8.49, *p* < 0.001], all favoring video telephonic counseling. Lastly, a tendency was observed for trust also in favor of video counseling [*F*_(2, 275)_ = 3.04, *p* = 0.05; Table 2].

**Table 2:** Comparison of counseling formats.

**Item**	**All contacts**	**Office**	**Phone**	**Video**	***df***	***F***	***p***
**Assessment of therapeutic contact**							
Global judgment conversation contact	1.5 ± 0.9	1.6 ± 1.0	1.9 ± 1.3	1.4 ± 0.7	2	3.39	**0.04**
Personal-impersonal	1.8 ± 1.0	1.7 ± 0.9	1.8 ± 1.0	1.8 ± 1.0	2	0.40	0.67
Pleasant-unpleasant	1.6 ± 0.9	1.7 ± 1.0	1.6 ± 0.9	1.5 ± 0.6	2	3.35	**0.04**
Friendly-unfriendly	1.3 ± 0.6	1.4 ± 0.7	1.4 ± 0.7	1.2 ± 0.4	2	5.55	**0.004**
Suitable-unsuitable	1.7 ± 1.0	1.7 ± 1.0	1.8 ± 1.3	1.6 ± 1.0	2	0.51	0.60
Helpful-not helpful	1.9 ± 1.1	1.9 ± 1.2	2.0 ± 1.3	1.8 ± 1.0	2	0.45	0.64
Understanding-not understanding	1.5 ± 0.9	1.6 ± 0.9	1.6 ± 1.0	1.4 ± 0.7	2	2.14	0.12
Supportive-not supportive	1.8 ± 1.0	1.8 ± 1.2	1.8 ± 1.0	1.7 ± 0.9	2	0.89	0.41
Empathic-not empathic	1.6 ± 0.9	1.8 ± 1.1	1.6 ± 0.9	1.5 ± 0.8	2	2.91	0.06
I felt comfortable-I felt uncomfortable.	1.7 ± 1.0	1.9 ± 1.2	1.7 ± 1.0	1.4 ± 0.7	2	8.49	** <0.001**
I could open up well-I could not open up well.	1.7 ± 1.0	1.9 ± 1.0	1.7 ± 1.0	1.6 ± 0.8	2	2.80	0.06
I fully trust my counselor/therapist-I cannot trust my counselor/therapist.	1.9 ± 1.1	2.1 ± 1.2	1.9 ± 1.0	1.8 ± 1.0	2	3.04	**0.05**
My concern and my problem situation were comprehensively inquired about and understood-my concern and my problem situation were insufficiently inquired about and understood.	1.8 ± 1.0	1.8 ± 1.1	1.8 ± 1.2	1.7 ± 0.9	2	0.70	0.50
**Therapeutic relationship**							
I believe that my counselor/therapist is helping me.	4.4 ± 1.6	4.4 ± 1.6	4.7 ± 1.2	4.4 ± 1.6	2	0.68	0.51
I believe that counseling is helping me.	4.4 ± 1.5	4.3 ± 1.6	4.8 ± 1.2	4.3 ± 1.5	2	1.19	0.30
I have gained some new insights.	4.0 ± 1.5	4.0 ± 1.6	4.0 ± 1.5	4.0 ± 1.5	2	0.01	0.99
I have recently started to feel better.	3.0 ± 1.4	2.7 ± 1.4	3.3 ± 1.6	3.2 ± 1.4	2	4.97	**0.008**
I can already foresee that I may be able to overcome the problems I came to counseling for.	3.3 ± 1.4	3.3 ± 1.5	3.6 ± 1.3	3.4 ± 1.4	2	0.52	0.59
I feel that I can rely on the counselor/therapist.	4.6 ± 1.4	4.6 ± 1.5	4.9 ± 0.9	4.6 ± 1.5	2	0.53	0.59
I feel that the counselor/therapist understands me.	4.7 ± 1.4	4.6 ± 1.4	4.7 ± 1.3	4.9 ± 1.3	2	0.75	0.47
I feel that the counselor/therapist wants me to achieve my goals.	5.0 ± 1.3	5.0 ± 1.3	5.2 ± 0.9	5.0 ± 1.4	2	0.19	0.83
I feel that I, as well as the counselor/therapist, are seriously pulling together.	4.6 ± 1.4	4.5 ± 1.4	5.0 ± 1.1	4.6 ± 1.4	2	1.24	0.29
I feel that I and the counselor/therapist see and assess my problems similarly.	4.5 ± 1.4	4.5 ± 1.5	4.7 ± 1.1	4.5 ± 1.3	2	0.52	0.60
I feel that I can now understand myself and deal with myself independently.	3.2 ± 1.5	3.2 ± 1.5	3.2 ± 1.6	3.2 ± 1.5	2	0.01	0.99
**Hurdles**							
The necessary technology/framework conditions overwhelmed me.	1.8 ± 1.3	1.7 ± 1.2	2.0 ± 1.7	1.8 ± 1.4	2	0.79	0.45
The necessary technology/framework conditions were very distracting to me.	1.6 ± 1	1.5 ± 0.8	1.6 ± 1.1	1.6 ± 1.2	2	1.48	0.23
I was able to fully concentrate on the content of the conversation.	4.7 ± 1.7	4.6 ± 2.2	4.7 ± 1.8	4.8 ± 1.7	2	0.37	0.69
I was worried about catching a cold.	1.2 ± 0.7	1.2 ± 0.7	1.3 ± 0.9	1.2 ± 0.8	2	0.05	0.95
I was worried about doing something wrong.	1.7 ± 1.2	1.8 ± 1.2	1.8 ± 1.2	1.7 ± 1.2	2	0.08	0.92

*Significant p values are displayed in bold*.

“Therapeutic relationship” was rated with mean item scores ranging from 2.7 to 5.2 (higher scores represent higher agreement, Table 2). The office subgroup rated the “therapeutic relationship” between 2.7 and 4.4. A similar picture emerged for the phone (3.3–4.8) and video (3.2–4.4) subgroups (Table 2). A significant difference was found between groups for the item: “I have recently started to feel better” [*F*_(2, 275)_ = 4.97, *p* = 0.008] favoring the phone and video contact, while for neither of the other items significant differences were observed (Table 2).

The “hurdles” were rated on average as low (1.8 ± 1.3) in the overall group when assessing the item “The necessary technology/framework conditions overwhelmed me.” The same picture emerged in all three subgroups: office (1.7 ± 1.2), phone (2.0 ± 1.7), and video (1.8 ± 1.4, *p* > 0.05). No significant differences were found between the subgroups for this or other items of the “hurdles” subject area (*p* > 0.05; Table 2).

A regression analysis showed no significant effect of age on “assessment of therapeutic contact” [age: *F*_(1, 277)_ = 0.18, *p* = 0.672]. However, a significant effect of age on the assessment of therapeutic contact as personal was shown by means of a regression analysis [*F*_(1, 277)_ = 4.50, *p* = 0.035] with higher age. This means that older persons perceive and experience the video intervention as partly more impersonal. Within the subordinated subject area “hurdles” a significant effect of age on the item “The necessary technology/framework conditions overwhelmed me” was detected using a regression analysis [*F*_(1, 277)_ = 7.85, *p* = 0.005] with higher age. Again, the results indicate that elders perceive the video call as more challenging. Lastly, a regression analysis showed a significant effect of age on the item “I was able to fully concentrate on the content of the conversation” [*F*_(1, 277)_ = 14.85, *p* < 0.001] with higher age. This is further evidence that older people may perceive the videophone format to be more impersonal and depersonalized. No significant gender effects were found using regression analyses.

## Discussion

The COVID-19 pandemic in 2020 was the basis for the need to establish digital structures in the medical consultation and therapy setting in order to ensure continuous psychosomatic care. Old structures had to be modified (e.g., wearing face masks during office counseling) and were augmented by phone and video formats. Here, the question arose whether digital counseling services (telephony or videotelephony) were equally accepted compared to the traditional face-to-face office setting.

In the context of this explorative study, an *ad hoc* questionnaire was used, which assessed the subject areas “patient characterization,” “assessment of therapeutic contact,” “therapeutic relationship,” and “hurdles.” Regarding the “therapeutic relationship” it was shown that all three counseling formats (office, telephony, video) were perceived as very positive. Despite the lack of physical presence in the digital formats, it was possible to establish a trusting and understanding therapeutic relationship. These results are in line with previous studies reporting the establishment of a functional therapeutic relationship that is perceived as appreciative and trusting (18–21). Accordingly, the therapeutic relationship can be built in the (video) telephonic setting as also suggested before (4, 21, 22).

Regarding “assessment of therapeutic contact” general acceptance and high satisfaction were found with all the counseling formats also in line with similar studies that report the “evaluation of the therapeutic contact” in both tele-counseling and face-to-face contact as satisfactory, trusting and appreciative (23, 24). Here it should be noted that the majority of the sessions in the study, were individual sessions and did not take place in the context of therapy. In the present study, the “assessment of therapeutic contact” could be manifested in online vs. face-to-face office setting, in addition to the already existing literature, also in psychosomatic medicine. Interestingly, the video group scored higher on global judgement, personal contact, pleasantness, friendliness, and feeling comfortable indicating that it was perceived positively to see the therapist again without a mask and be able to make full visual contact. In contrast, the consultation session via video or phone in times of the covid-19 pandemic reduced the possibility of contracting COVID-19, which made many patients feel safer. The written free-text modules corroborated the assumption that a protected space can also be created within digital offerings, which offers the patient the opportunity to “arrive” from home, to gain trust and to engage in the therapeutic relationship. Some examples from the free text modules are: “One of my problems is panic attacks when driving somewhere, so it was a good idea to have the conversation over the phone,” or: “Distancing rules can be adhered to without any problems.”, “Video call: risk minimization, time expenditure, environment.”, “No travel hassle and no waiting.” Nevertheless, it was also found through free text modules that many patients very much appreciate face-to-face office contacts and the associated complexity of the conversation. Some comments from the free text modules read: “On site I feel more undisturbed and am not so quickly distracted,” “Conversation is more personal and therefore problems are easier to address,” “Contact on site is best because you can explain and present your issues better.” Lastly, as shown also in previous studies (25, 26) there is a great acceptance and broad agreement regarding tele-counseling in both men and women.

In terms of “hurdles” the comparison did not detect differences between the three counseling formats. However, it is to note that a significant age difference was found for the item “The necessary technology/framework conditions overwhelmed me.” It can be assumed here that in middle to increasing age, technical hurdles and complications could lead to feelings of being overwhelmed and to impairment of the consultation/therapy conversation. Prior research also suggested that with increasing age the tolerance level regarding technical hurdles or complications decreases and consequently the video contact cannot be fully utilized and may be perceived as unsatisfactory (27). However, despite potential difficulties and technical hurdles, regular use and greater support could reduce technical hurdles (3, 28). Another age effect was found for the hurdles item “I was able to fully concentrate on the content of the conversation.” With increasing age, the patients indicated that they were less able to concentrate, especially in phone conversations. This suggests that the visual aspect, whether digitally by means of the camera or face-to-face in the office, is likely to represent an important factor, which can be perceived as a hurdle when omitted.

In consideration of the fact that there is already some evidence for the acceptance and effectiveness of video-based and phone counseling formats (10, 13, 22, 23, 27), this exploratory study was able to show that especially in psychosomatic counseling, video-telephonic counseling can represent an essential and effective support function. As possible limitations, it should be noted that the present study was based on a single time point exploratory online survey in psychosomatic patients without follow up and without healthy control group. Therefore, generalization should be performed with caution. Generalizability is limited because it cannot be concluded from the data how many patients were eligible for this study. Accordingly, no differences in patient characteristics between participants and non-participants can be drawn. Nonetheless, the strength of the study is the assessment in the height of the pandemic under real life naturalistic conditions assessing the acceptance of different counseling formats during times of high need. It is essential that further research focuses on how the availability and evaluation of digital counseling services changes over the course of the pandemic and beyond.

In summary, the present pilot study shows that digital consultation formats were perceived as a promising addition to the traditional face-to-face office consultation setting. Digital formats (phone, video) were not generally preferred to face-to-face office counseling in practice, but video counseling was accepted and perceived with great satisfaction and acceptance and rated higher in terms of pleasantness and feeling comfortable, possible due to the pandemic conditions. Another point that should be noted is the professional background of the treating therapist, which could have an influence on the satisfaction of the patient and should therefore be investigated in further studies. In the future, online based formats could also represent an additional care structure in the medical counseling setting (1, 29). Future studies should therefore examine tele-counseling from the therapist's perspective and look more closely at long-term therapies in a digital setting. It is very likely that tele-counseling and especially video counseling will persist in the post COVID-19 era and remain/become an important complement to traditional psychosomatic office counseling formats.

## Data Availability Statement

The raw data supporting the conclusions of this article will be made available by the authors, without undue reservation.

## Ethics Statement

The study was approved by the local Ethics Committee (458/2020BO). The patients/participants provided their written informed consent to participate in this study.

## Author Contributions

JL performed the data analysis and wrote the first draft of the manuscript. AS, NS, and SZ planned and initiated the study. AS conducted the data generation and gave critical input throughout the study. All authors finalized the manuscript and contributed to the article and approved the submitted version.

## Conflict of Interest

The authors declare that the research was conducted in the absence of any commercial or financial relationships that could be construed as a potential conflict of interest.

## Publisher's Note

All claims expressed in this article are solely those of the authors and do not necessarily represent those of their affiliated organizations, or those of the publisher, the editors and the reviewers. Any product that may be evaluated in this article, or claim that may be made by its manufacturer, is not guaranteed or endorsed by the publisher.
